# Serum Levels of Inflammatory and Fibrotic Cytokines in Patients with Carpal Tunnel Syndrome and Hip Osteoarthritis

**DOI:** 10.3390/biomedicines11010011

**Published:** 2022-12-21

**Authors:** Mirjana Baričić, Olga Cvijanović Peloza, Ana Terezija Jerbić Radetić, Veljko Šantić, Hrvoje Omrčen, Sanja Zoričić Cvek

**Affiliations:** 1 Clinical Orthopaedic Hospital Lovran, 51415 Lovran, Croatia; 2 Department of Anatomy, Faculty of Medicine, University of Rijeka, 51000 Rijeka, Croatia

**Keywords:** carpal tunnel syndrome, hip osteoarthritis, TGF-β1, BMP-7, IL-1β, TNFα

## Abstract

A certain percentage of carpal tunnel syndrome (CTS) is associated with inflammatory conditions. Osteoarthritis (OA) increases the risk of CTS, and both diseases are common in the general population. Moreover, OA and CTS are often present in the same patients. Since inflammation and fibrosis are found in both conditions, the question is whether circulating inflammatory cytokines and cytokines involved in fibrosis in OA and CTS patients could serve as indicators of coexisting CTS and OA pathology. This investigation was performed on 31 CTS patients, 29 hip OA patients, and 15 healthy volunteers. Blood samples were collected, and serum levels of TGF-β1, BMP-7, IL-1β, and TNFα were measured using the ELISA method. The statistical analysis was performed to reveal the most significant differences in the serum levels of these cytokines. Statistical significance was set at *p*-values ≤ 0.05. The serum level of TGF-β1 was the highest in CTS patients (16.36 pg/mL) and significantly different compared to OA and healthy control. Analysis of the cytokine serum level in the subdivided group revealed that serum levels of TGF-β1 and BMP-7 were significantly higher in CTS+/OA+ patients as well as BMP-7 in the OA+/CTS+ group. There was no significant difference in serum levels of the inflammatory cytokines TNFα and IL-1β among all groups. This study showed that in the end stage of CTS and OA, serum levels of inflammatory cytokines (IL1-β and TNFα) were not altered, while the serum levels of TGF-β1 and BMP-7 were significantly higher, especially in patients with coexisting OA and CTS. These findings suggest the possible values of TGF-β1 and BMP-7 as a predictive factor for the comorbidity of CTS and OA.

## 1. Introduction

Carpal tunnel syndrome (CTS) is the most common compressive neuropathy of the upper extremities caused by compression and/or traction of the median nerve at the carpal tunnel level. The increased pressure in the carpal tunnel gradually decreases microcirculation, causes edema and inflammation, and finally, at the end stage of the disease, causes subsynovial tissue hypertrophy and fibrosis [1,2,3]. Besides all other changes, the compression of the median nerve results in inflammation of the myelin sheath, and consequently, nerve fibers undergo atrophy and infarction. At the end stage of CTS, the most common pathohistological finding is progressive fibrosis of the subsynovial connective tissue (SSCT) [4]. Since inflammation is the main pathophysiological process during the onset of CTS, it has been presumed that molecular factors such as inflammatory cytokines are the key regulators of CTS [5,6]. Also, due to end-stage fibrosis, it could be presumed that fibrotic factors such as TGFβ are involved in the pathophysiological mechanism underlying CTS [7,8,9,10]. 

Although most CTS are idiopathic disorders, CTS can be associated with metabolic and inflammatory conditions such as diabetes mellitus, hypothyroidism, and connective tissue disease (CTD), including rheumatic disorders [2,11]. Extensive meta-analyses from 2016 by Shiri et al. [12] and from 2003 by van Dijk et al. [13] reported that RA and OA increased the risk of CTS. According to their investigation, a two-fold increased risk of CTS was found in patients who suffer from both types of arthritis, and they suggested that the same predicts the development of CTS, although the underlying mechanism for that may be different. Furthermore, it is suggested that RA may increase the risk of CTS by causing a local inflammatory process of the tendons and tendon sheaths, while OA is related to bony hypertrophy and increased pressure inside the carpal tunnel. Since OA also causes local synovial membrane inflammation, it can be hypothesized that the inflammatory factors released in circulation could potentially cause pathological changes in the distant skeletal structures.

OA is the most frequent degenerative disease of synovial joints. It is primarily characterized by degeneration and destruction of articular cartilage, but changes also affect periarticular tissue, synovial membrane, and subchondral bone. Along with degeneration of articular cartilage, osteophyte development, subchondral sclerosis, and chronic synovitis are also present. OA is a chronic arthropathy that is associated in its early stages with inflammation of the synovial tissue [14,15]. In this context, different pro-inflammatory cytokines such as IL-1β, TNFα, IL-6, IL-15, IL-17, and IL-18 [15,16,17] are reportedly involved in the pathophysiology of OA. Among them, IL-1β and TNFα are considered key inflammatory cytokines involved in the pathophysiological process of OA. OA patients have increased IL-1β levels and their receptors in synovial membrane and synovial fluid, cartilage, and subchondral bone [15]. 

As a part of the cytokine network regulating processes during tissue damage and regeneration, BMPs and TGFβ are stressed as the most potent regenerative and protective growth factors but are also involved in fibrosis [18,19] and osteophyte formation. TGF-β1 is a TGFβ isoform primarily involved in the induction of fibrosis [16], and it has been shown that it is overexpressed in the subsynovial tissue of CTS patients and experimental models of CTS (8). On the other side, BMP-7 is an important morphogenetic factor that induces regenerative processes in damaged tissue and can also protect damaged organs from fibrosis [17]. 

So far, published studies report OA as a risk factor for the development of CTS related to the basal joint arthritis of the thumb, cervical arthritis, and trigger digit [14], but there is no relevant literature data that relate OA of large joints (hip and knee OA) with the incidence of CTS. Because the coexistence of these diseases is common in the general population, the question is whether CTS may be present in an OA patient and regulated by the same molecular mechanism. Thus far, no studies have been conducted dealing with this issue. 

Due to the inflammatory and fibrotic pathophysiological background of both OA and CTS, the strong association of inflammatory and fibrotic cytokines secreted locally and in serum with these two skeletal diseases, and the frequent coexistence of these two diseases in the same patient, it could be hypothesized that there are circulating factors connecting these two entities.

Our study investigated the serum levels of two pro-inflammatory cytokines, TNFα and IL-1β, both of which are proven to be strongly connected with the inflammation of synovial membranes in OA joint and SSCT in CTS. Also, we investigated serum levels of two fibrotic factors, TGF-β1 and BMP-7, which are regulators of regenerative processes of skeletal tissues and/or fibrotic processes. The main goal was to discern which of these factors best characterized the patients with coexisting CTS and hip OA. Therefore, we aimed to compare the serum concentration of pro-inflammatory and fibrotic factors in patients diagnosed with single OA or CTS and patients with coexisting OA and CTS. We believe that the results of this investigation will give us better insight into the association between OA and CTS and determine the possible role of these cytokines in predicting the coexistence of these diseases. The main question is whether these factors could be a screening tool in patients with idiopathic CTS.

## 2. Materials and Methods

### 2.1. Participants and Clinical Assessment 

For this study, 60 participants were recruited from the patients registered in the Clinical Orthopedic Hospital Lovran, Faculty of Medicine, University of Rijeka, from September 2021 to January 2022, due to a planned surgical procedure either for total hip replacement surgery or for decompression of the carpal tunnel. 

A group of 31 participants undergoing surgical treatment for carpal tunnel decompression was designated as the CTS group. Diagnosis was confirmed using anamnestic data (hand pain and finger tingling), clinical signs and symptoms (hypesthesia in the median nerve (MN) region, hypotrophy/atrophy of the thenar muscle, positive Tinel and Phalen tests), and sensory and motor impairment of the median nerve (ENG, Medelec Sinergy Multimedia, EMG/EP system, Viasys Healthcare) according to the criteria of the American Academy of Neurology [20], and were evaluated as the third stage of the disease [21,22]. Exclusion criteria were diabetes mellitus, obesity, hyperuricemia, amyloidosis, systemic lupus erythematosus, rheumatoid arthritis, kidney failure, trauma and malignancy, a history of radiculopathy and neuropathy, and previous surgery. Due to their participation in this study, these patients were subjected to additional clinical examination to reveal the coexistence of hip OA. According to the additional tests, 17 patients were diagnosed with CTS, and 14 patients were diagnosed with the coexistence of CTS and moderate stage of hip OA.

A group of 29 patients undergoing hip replacement surgery in our hospital was designated as the OA group. The diagnosis was confirmed by clinical and radiological criteria according to WOMAC (Western Ontario and McMaster Universities Osteoarthritis Index) and the American College of Rheumatology [23,24]. Exclusion criteria were autoimmune, inflammatory, and metabolic diseases, trauma and malignancy, and previous surgery. These patients were also subjected to additional clinical and electrophysiological examinations to reveal if some of them had CTS symptoms. The clinical criteria for CTS were the same as described above. In this group, 15 patients were diagnosed with OA, and 14 patients were diagnosed with OA and moderate CTS. 

Also, 15 volunteers were recruited for the healthy control group after being checked for medical history and the presence of clinical signs and symptoms for CTS or OA. 

All participants included in this study signed an informed consent form. This study was conducted according to the ethical principles of the Declaration of Helsinki and was approved by the Ethics Committee of the Faculty of Medicine, University of Rijeka and Clinical Orthopedic Hospital Lovran, Croatia. This study was also approved by the Institutional Scientific Board and is a part of extensive research of a medical doctor’s doctoral thesis.

### 2.2. Blood Sampling and Cytokine Assay

Blood samples (5 mL) were collected under controlled conditions (nonfasting state, 8 am to 9 am, the day or two before planned surgical treatment). Upon centrifugation (3000× *g* rpm, 10 min), serum aliquots were removed and stored in plastic tubes at −20 °C until assayed. Serum samples were identified by random numbers, and the demographic and clinical data were matched with the same identification number. All data were known to a person who did not participate in this study. 

Prior to analysis, sera samples were thawed at room temperature and mixed slowly. The serum concentration of IL-1β and BMP-7 were measured with sandwich enzyme-linked immunosorbent assay (human IL-1β ELISA kit DLB50; human BMP-7 ELISA kit DBP700, R&Dsystems, Minneapolis, MN, USA), and TGF-β1 and TNFα were measured with sandwich enzyme-linked immunosorbent assay (TGF-β1 human ELISA kit, ab 100647, human TNFα simple step ELISA kit ab181421, Abcam, Cambridge, MA, USA), respectively. The analyses were performed according to the manufacturer’s instructions. In line with this, a standard curve for each biomarker was created from the mean absorbance of specific standard dilutions performed in duplicate and their respective concentrations. Absorbances at 450 nm (450 nm as measuring wavelength) were determined with a microplate reader (Euroimmun analyzer I-2P, Lubeck, Germany). Concentrations of the sample proteins were read from the standard curves. The Human Interleukin-1β (IL-1β) ELISA kit had an assay range of 3.9 to 250 pg/mL and a sensitivity of 1 pg/mL. The Human Bone Morphogenetic protein-7 (BMP-7) ELISA kit had an assay range of 31.2 to 2000 pg/mL and a sensitivity of 7.83 pg/mL. The Human Transforming Growth Factor-β1 (TGF-β1) ELISA kit had an assay range of 18 to 4000 pg/mL and a sensitivity of 80 pg/mL. The Human Tumor Necrosis Factor -α (TNF-α) ELISA kit had an assay range of 15.63 to 1000 pg/mL and a sensitivity of 4.32 pg/mL. The average intra- and interassay coefficients of variation were <10% for all biomarkers. The ELISA method was performed at the Department of Chemistry and Biochemistry, Faculty of Medicine, University of Rijeka and at the Biochemistry Laboratory of the Clinical Hospital Rijeka by two independent researchers. 

### 2.3. Statistical Analysis

Data analysis was performed using Statistica, version 14.0. (TIBCO Software Inc.). Categorical data were compared with a Pearson Chi-squared test. The normality of quantitative data was tested with the Kolmogorov–Smirnov test, which showed that the distribution was not normal. The quantitative data are presented as the median and range. Mann–Whitney U-test and Kruskal–Wallis ANOVA tests were used to test differences between quantitative variables that were not normally distributed. After ANOVA, we used the post-hoc Scheffe test. *p*-values lower than 0.05 were considered statistically significant. 

## 3. Results

### 3.1. Demographic Data

The demographic data of 60 patients and 15 healthy control subjects recruited for this study are shown in Table 1. An approximately equal number of male and female patients was included in the study, with a median age of 60 to 70. The body mass index (BMI) was calculated based on the measured height and weight. Fourteen patients showed higher BMI than normal, but these patients were not clinically obese. A minor number of patients reported possible occupation risk, but it was not connected to their profession. The CRP level was normal in all groups of participants in this study: the mean level of CRP was 4.6 (±1.63) mg/L in the CTS+/OA− group, 3.6 (±1.34) mg/L in the CTS+/OA+ group, 4.6 (±1.86) mg/L in the OA+/CTS− group, and 3.8 (±1.39) mg/L in the OA+/CTS+ group. In the control group of patients, the CRP level was 3.9 (±1.33) mg/L. 

### 3.2. Serum Levels of Inflammatory and Fibrotic Cytokines in CTS, OA, and Healthy Control Groups

The analysis of BMP-7, IL-1β, and TNFα serum levels in CTS, OA, and control groups revealed no statistically significant difference, while the baseline TGF-β1 serum level differed and was significantly higher in the CTS group of patients (median value 16.36 pg/mL vs. 3.26 pg/mL in OA and 2.6 pg/mL in the control group, respectively; *p* < 0.0001). The median, upper, and lower quartiles as well as Mann–Whitney U-test results for comparison between different groups of patients are presented in Table 2.

### 3.3. Serum Levels of Inflammatory and Fibrotic Cytokines in CTS/OA+ and OA− Subgroups and OA/CTS+ and CTS− Subgroups 

The CTS group of patients was divided into two subgroups regarding the coexistence of OA. The analysis of cytokine serum levels revealed statistically significant differences for BMP-7 and TGF-β1, while TNFα and IL-1β serum levels did not differ significantly. 

Also, the OA group was divided into two subgroups regarding the coexistence of CTS. The analysis showed that the BMP-7 serum level was significantly higher in patients diagnosed with both OA and CTS, while others did not differ significantly (Table 3). 

## 4. Discussion

The growth factors and cytokines are important factors involved in the regulation of tissue growth and development, tissue homeostasis, and the pathogenesis of various pathological processes such as inflammation, fibrosis, and neoplasms. Data regarding serum levels of certain inflammatory and/or fibrotic cytokines during the pathogenesis of various skeletal diseases could serve as a prognostic indicator and screening factor for accompanying diseases. 

In this study, we analyzed the serum levels of TNFα and IL-1β as the most important pro-inflammatory cytokines and TGF-β1 and BMP-7 as growth factors involved in tissue regeneration and fibrosis. We were interested in whether these factors could indicate the coexisting skeletal disease in the case of CTS and/or hip OA. 

Our study results showed that the TNFα and IL-1β serum levels in CTS and OA patients did not differ significantly, even compared with healthy controls. These results are in line with the study by Karimi et al. from 2020, which showed no difference in TNFα, IL-1β, IL-6, and IL-10 serum levels between CTS patients and healthy controls [25], as well as with the results of Freeland et al. [5], although some reports showed increased levels of other inflammatory cytokines [26]. Pro-inflammatory cytokines are critical mediators of tissue destruction, and so far, they seem to be the main cytokines involved in local processes of joint destruction during OA. IL-1β is elevated in synovial fluid, synovial membrane, cartilage, and subchondral bone [27]. Since inflammation is usually present in the early stage of both CTS and OA, our results could be explained by the fact that the patients included in this study were in the late stage of the disease in which the inflammation is not present anymore. It could be hypothesized that the inflammatory cytokines are critical for the onset of CTS and OA, and after the destruction of joint tissues is completed, the regenerative processes start. During the OA process, the production of various inflammatory cytokines can vary depending on the duration and severity of the disease [28,29,30]. 

Therefore, the cytokines involved in tissue fibrosis included in the analysis showed that the TGFβ and BMP-7 serum levels were significantly elevated in the CTS group of patients compared with OA patients and healthy controls. This can be explained by the fact that the most common and prominent local findings in CTS patients, especially at the end stage of the disease, is fibrosis of the subsynovial tissue [4]. 

The most interesting were the results of the analysis of the serum level of the cytokines involved in fibrosis in CTS and OA patients subdivided into two subgroups according to the presence of coexisting disease. These results showed that serum levels of both these cytokines, TGF-β1 and BMP-7, were significantly higher in patients with the coexistence of CTS and OA compared with CTS patients, while in the OA/CTS+ patients, only BMP-7 was higher compared with OA patients. These results suggest that, at least in some OA patients, data related to the elevated TGF-β1 and BMP-7 serum levels could predict the comorbidity of CTS. Generally, it is hypothesized that the balance between TGF-β1 and BMP-7 is important for maintaining the extracellular matrix and that disruption of this balance toward TGFβ leads to fibrogenesis in various organs. It is confirmed that BMP-7 counteracts TGFβ activity during fibrinogenesis and can inhibit epithelial-to-mesenchymal transition and therefore decrease the number of fibroblasts involved in fibrosis [31,32]. 

Our study showed no relevant importance of TNF and IL-1β serum levels in patients at the end stages of OA and CTS. Importantly, elevated TGF-β1 and BMP-7 serum levels in the same patients, especially in patients with coexisting diseases, could suggest their importance in the pathogenesis of these diseases and the possible use of these molecular factors in screening for comorbidity. Nevertheless, further studies with a larger sample size and more specific screening of patients included in the investigation of TGF-β1 and BMP-7 should be performed to confirm our hypothesis. 

## Figures and Tables

**Table 1:** Demographic data.

	CTS+/OA− Group	CTS+/OA+ Group	OA+/CTS− Group	OA+/CTS+ Group	Control Group	*p*-Value
*N*	17	14	15	14	15	
Gender						
M	9	8	10	6	12	0.29
F	8	6		5	3	
Age (year)						
median	59.5	73	60	71	60	<0.00 *
(range)	(48–81)	(54–85)	(50–75)	(47–83)	(60–64)	
BMI						
mean (±st. dev.)	30.7	32.3	33.7	34.1	31.3	0.10
	±1.5	±1.5	±2.6	±2.8	±1.3	
Occupation risk						
no risk	14	10	14	12	15	0.20
possible risk	3	4	1	2	0	

* statistically significant difference.

**Table 2:** Serum levels of inflammatory and fibrotic cytokines in CTS and OA groups of patients and healthy controls.

Cytokine (pg/mL)	CTS (*N* = 31)	OA (*N* = 29)	Control (*N* = 15)	Sheffe Test *p*-Value
BMP-7	1.4 (0.3–2.6)	0.2 (0.1–4.17)	1.0 (0.12–2.0)	0.28
IL-1β	0.43 (0.29–0.9)	0.58 (0.29–1.0)	0.92 (0.56–1.9)	0.29
TGF-β1	16.36 (10.0–22.7)	3.26 (0.25–6.4)	2.6 (0.25–4.9)	0.00 *
TNFα	4.81 (4.14–6.23)	5.7 (4.46–7.6)	4.9 (4.24–5.27)	0.41

* statistically significant difference. The results are expressed as a median (25th and 75th percentiles).

**Table 3:** Serum levels of inflammatory and fibrotic cytokines in CTS and OA groups of patients subdivided regarding the coexistence of skeletal diseases.

Cytokine (pg/mL)	CTS+/OA− *N* = 17	CTS+/OA+ *N* = 14	*p*-Value	OA+/CTS− *N* = 15	OA+/CTS+ *N* = 14	*p*-Value
BMP-7	0.50 (0.20–2.50)	2.60 (0.80–4.50)	0.03 *	0.11 (0.10–0.20)	3.20 (0.32–4.80)	0.01 *
IL-1β	0.72 (0.43–1.15)	0.43 (0.29–0.50)	0.31	0.35 (0.29–0.86)	0.72 (0.43–1.29)	0.14
TGF-β1	10.96 (9.60–12.60)	22.74 (20.3–38.23)	0.00 *	1.78 (0.23–22.70)	4.37 (1.51–6.43)	0.50
TNFα	4.87 (4.14–6.23)	4.77 (4.52–5.69)	0.77	5.08 (3.96–5.90)	5.90 (5.00–8.02)	0.09

* statistically significant difference. The results are expressed as a median (25th and 75th percentiles).

## Data Availability

Not applicable.
